# Supplementary material for: Extracts from Wallis Sponges Inhibit Vibrio harveyi Biofilm Formation
Source: Microorganisms. 2023 Jul 6;11(7):1762. doi: 10.3390/microorganisms11071762 (PMC10383632; doi:10.3390/microorganisms11071762)
Supplement: Supplementary file 1 [file microorganisms-11-01762-s001.zip › microorganisms-2470603-supplementary.pdf]

Table S1. Queensland Museum (QM) accession numbers, species and corresponding OTUs

Reference	QM Registration	Genus species	OTU
P559	G339004	*Petrosia sp.*	QM2721
P560	G339005	*Cinachyrella sp.*	QM4680
P561	G339006	*Echinodictyum asperum*	QM0133
P562	G339007	*Suberea laboutei*	QM1511
P563	G339008	*Stylissa cf. carteri*	QM0336
P564	G339009	*Myrmekioderma sp.*	QM4997
P571	G339016	*Dendrilla sp.*	QM2575
P572	G339017	*Dysidea lizardensis sp.*	QM1519
P573	G339018	*Aplysilla sp.*	QM2034
P574	G339019	*Haliclona (Haliclona) sp.*	QM4999
P575	G339020	*Haliclona (Haliclona) sp.*	QM4499
P579	G339024	*Stylissa massa*	QM0925
P581	G339026	*Hyrtios cf. erectus*	QM0796
P582	G339027	*Pericharax heteroraphis*	QM0668
P586	G339031	*Acanthodendrilla cf. 1948*	QM1948
P587	G339032	*Hyrtios erectus*	QM0796
P588	G339033	*Axinyssa sp.*	QM3251
P589	G339034	*Oscarella sp.*	
P590	G339035	*Fascaplysinopsis sp.*	QM1549
P593	G339038	*Leiosella sp.*	QM6001
P594	G339039	*Hyrtios erectus*	QM0796
P595	G339040	*Hyrtios erectus*	QM0796
P596	G339041	*Psammocina sp.*	QM1944
P597	G339042	*Rhaphoxya pallida*	QM0465
P600	G339045	*Jaspis sp.*	QM4187
P601	G339046	*Stylissa cf. carteri*	QM0922
P602	G339047	*Haliclona (Haliclona) sp.*	QM1971
P603	G339048	*Pseudoceratina sp.*	QM1947
P605	G339050	*Leucetta chagosensis*	QM1402
P607	G339052	*Halichondrida (Halichondrida) sp.*	QM1429
P608	G339053	*Ircinia sp.*	QM1244
P609	G339054	*Pericharax sp.*	QM1361 or QM2065
P616	G339061	*Astrosclera willeyana*	QM0656
P618	G339063	*Pericharax sp.*	QM2116
P620	G339065	*Petrosia sp.*	QM4179
P621	G339066	*Petrosia sp.*	QM2035
P622	G339067	*Lissodendoryx (Ectydoryx) sp.*	QM1281
P625	G339070	*Fascaplysinopsis sp.*	QM6004
P628	G339073	*Stylissa cf. carteri*	QM0922
P633	G339078	*Dysidea cf. pallescens sp.*	QM0630
P634	G339079	*Halichondrida (Halichondrida) sp.*	QM1984
P636	G339081	*Suberea sp.*	QM2121
P640	G339085	*Ircinia sp.*	QM2707
P642	G339087	*Cacospongia sp.*	QM6009
P646	G339091	*Fascaplysinopsis sp.*	
P658	G339103	*Chelonaplysilla delicata*	QM1829
P660	G339105	*Petrosia sp.*	QM1895
